# Metagenomic Assembly and Prokaryotic Metagenome-Assembled Genome Sequences from the Northern Gulf of Mexico “Dead Zone”

**DOI:** 10.1128/MRA.01033-18

**Published:** 2018-09-06

**Authors:** J. Cameron Thrash, Brett J. Baker, Kiley W. Seitz, Ben Temperton, Lauren Gillies Campbell, Nancy N. Rabalais, Bernard Henrissat, Olivia U. Mason

**Affiliations:** a Department of Biological Sciences, Louisiana State University, Baton Rouge, Louisiana, USA; b Marine Science Institute, University of Texas, Austin, Port Aransas, Texas, USA; c School of Biosciences, University of Exeter, Exeter, United Kingdom; d Department of Earth, Ocean, and Atmospheric Sciences, Florida State University, Tallahassee, Florida, USA; e Department of Oceanography and Coastal Sciences, Louisiana State University, Baton Rouge, Louisiana, USA; f Louisiana Universities Marine Consortium, Chauvin, Louisiana, USA; g Architecture et Fonction des Macromolécules Biologiques, CNRS, Aix-Marseille Université, Marseille, France; h INRA, USC 1408 AFMB, Marseille, France; i Department of Biological Sciences, King Abdulaziz University, Jeddah, Saudi Arabia; University of Arizona

## Abstract

Coastal regions experiencing declining dissolved oxygen are increasing in number and severity around the world. However, despite the importance of microbial metabolism in coastal hypoxia, few metagenomic surveys exist. Our data set from within the second largest human-caused hypoxic region provides opportunities to more deeply explore the microbiology of these systems.

## Abstract (precis)

Coastal regions experiencing declining dissolved oxygen are increasing in number and severity around the world. However, despite the importance of microbial metabolism in coastal hypoxia, few metagenomic surveys exist.

## ANNOUNCEMENT

Marine systems suffering from declines in dissolved oxygen (DO) are becoming more numerous across the globe (1). Many coastal regions experience hypoxia (DO, ≤2 mg liter^−1^) due to eutrophication from farmland runoff, stratification, and a resulting cascade of microbial processes that consume DO. The northern Gulf of Mexico experiences seasonal bottom water hypoxia that can exceed 22,000 km^2^ (http://www.noaa.gov/media-release/gulf-of-mexico-dead-zone-is-largest-ever-measured), impacting fisheries and other coastal industries (2). To better understand the microbiology of this system, we sequenced bacterioplankton metagenomes from the 2013 hypoxic region. We previously reported the metabolic contributions from members of uncultivated groups of bacterioplankton (3); however, considerable sequence information for other taxa remains unanalyzed. Here, we report the unbinned metagenomic coassembly of six samples and 50 bacterioplankton metagenome-assembled genome (MAG) sequences.

Site selection, marine chemistry metadata, and extraction, sequencing, and assembly methods were previously described (3, 4). Briefly, metagenomes from the six sites were obtained using one lane of an Illumina HiSeq 2000 instrument (100-bp paired-end sequencing generated by Argonne National Laboratory). Coassembly of all six samples was performed using IDBA-UD (5), resulting in 28,028 contigs of ≥3 kbp (303 contigs were >50 kbp, 72 were >100 kbp, and the largest was 494,909 bp). Integrated Microbial Genomes with Microbiome Samples (IMG/G) (6) annotation predicted 220,893 and 3,176 protein-coding and RNA genes, respectively. Separately, we binned contigs into MAGs (3). The 50 MAGs reported here are distributed in the following taxonomic affiliations (see the Methods section in reference 3): *Actinobacteria* (*n* = 12), *Alphaproteobacteria* (*n* = 5), *Bacteroidetes* (*n* = 6), *Gammaproteobacteria* (*n* = 3), *Gemmatimonidetes* (*n* = 1), *Ignavibacteriae* (*n* = 2), *Nitrospina* (*n* = 5), *Planctomycetes* (*n* = 7), *Proteobacteria* (*n* = 1), *Synechococcus* (*n* = 3), *Verrucomicrobia* (*n* = 3), and unclassified (*n* = 2). Twenty-four of the MAGs were estimated at >50% complete, with 16 estimated at >75% complete based on CheckM (7) (see Table S1 at https://doi.org/10.6084/m9.figshare.6911729.v1). All but 2 MAGs had estimated contamination of <9%, with 40 having estimated contamination of <3%. While several of our *Actinobacteria* MAGs grouped with sequences near the important OM1 clade of marine *Actinobacteria* (8–10), these are not expected to be true OM1 organisms (see Figure S1 in reference 3 and Table S1 at https://doi.org/10.6084/m9.figshare.6911729.v1), but make useful references for future studies of the group.

Metabolic reconstruction and carbohydrate-active enzyme (CAZyme) prediction were also completed as described (see reference 3 and Table S1 at https://doi.org/10.6084/m9.figshare.6911729.v1). *Nitrospina* MAGs were the only ones with predicted nitrite-oxidizing metabolism, matching observations from 2012 (11). While the majority of MAGs encoded aerobic metabolism, several taxa additionally had partial to complete pathways for dissimilatory nitrate and/or sulfate reduction (*Rhodospirillales* and *Polaribacter*), including dissimilatory nitrate reduction to ammonium, and a few had predicted capacity for sulfur lithotrophy and possible autotrophy (*Chromatiales*, *Rhodospirillales*, and *Donghicola*) (see Table S1 at https://doi.org/10.6084/m9.figshare.6911729.v1). Future comparisons of these data with those from other low-DO systems will illuminate common functional features associated with hypoxia and also provide information about biogeographic distinctions among taxa associated with these regimes.

### Data availability.

The shotgun sequence data are available the NCBI Sequence Read Archive (SRA) database under the accession numbers SAMN05791315 to SAMN05791320, which comply with MIxS standards (12). The annotated contigs of ≥3 kbp are publicly available at IMG/M under organism ID (OID) 3300003894. The MAGs are publicly available at IMG/M under the following OIDs: 2651870013 to 2651870024, 2651870028 to 2651870030, 2651870044 to 2651870048, 2651870056, 2651870059 to 2651870064, 2651870067 to 2651870071, 2651870074 to 2651870082, 2651870084, 2651870085, 2651870087, 2651870089, 2693429794 to 2693429796, 2693429800, and 2693429806. For a spreadsheet containing tabs that detail CheckM results, taxonomy, IMG/M information, metabolic reconstructions, and transporter and CAZy predictions, see Table S1 at https://doi.org/10.6084/m9.figshare.6911729.v1.
